# Supplementary material for: Comparative multiomics analysis of cell physiological state after culture in a basket bioreactor
Source: Sci Rep. 2022 Nov 23;12:20161. doi: 10.1038/s41598-022-24687-4 (PMC9686226; doi:10.1038/s41598-022-24687-4)
Supplement: Supplementary file 1 — Supplementary Information 1. [file 41598_2022_24687_MOESM1_ESM.zip › raw data/Transcriptomic raw data/6.Enrichment/1.GO/TE1vsTC1/GSEA/TE1/PROTEIN_TARGETING/PROTEIN_TARGETING(GO_0006605).html]

Details for gene set PROTEIN\_TARGETING(GO:0006605)[GSEA]


|  || Dataset | readcount.sample |
| Phenotype | sample.cls |
| Upregulated in class | TE1 |
| GeneSet | PROTEIN\_TARGETING(GO:0006605) |
| Enrichment Score (ES) | 0.51097894 |
| Normalized Enrichment Score (NES) | 1.7750667 |
| Nominal p-value | 0.0 |
| FDR q-value | 0.21235976 |
| FWER p-Value | 0.494 |
Table: GSEA Results Summary

  

Fig 1: Enrichment plot: PROTEIN\_TARGETING(GO:0006605)
  
Profile of the Running ES Score & Positions of GeneSet Members on the Rank Ordered List

  

| PROBE | DESCRIPTION   (from dataset) | GENE SYMBOL | GENE\_TITLE | RANK IN GENE LIST | RANK METRIC SCORE | RUNNING ES | CORE ENRICHMENT || 1 | 103222840 | LOC103222840 |  |  | 441 | Infinity | -0.0194 | Yes |
| 2 | 103231257 | LOC103231257 |  |  | 1095 | 3.305 | 0.0442 | Yes |
| 3 | 103240797 | LOC103240797 |  |  | 1560 | 2.400 | 0.0908 | Yes |
| 4 | 103236180 | LOC103236180 |  |  | 1731 | 2.265 | 0.1468 | Yes |
| 5 | 103232300 | LOC103232300 |  |  | 1824 | 2.207 | 0.2046 | Yes |
| 6 | 103229165 | AKAP5 |  |  | 2488 | 1.860 | 0.2272 | Yes |
| 7 | 103235715 | SRP72 |  |  | 3021 | 1.701 | 0.2512 | Yes |
| 8 | 103226057 | SEC61G |  |  | 3120 | 1.682 | 0.2940 | Yes |
| 9 | 103240726 | AKAP12 |  |  | 3572 | 1.596 | 0.3187 | Yes |
| 10 | 119619579 | SRP9 |  |  | 3576 | 1.596 | 0.3633 | Yes |
| 11 | 103230793 | TOMM20 |  |  | 3669 | 1.580 | 0.4035 | Yes |
| 12 | 103244343 | SRP19 |  |  | 4184 | 1.505 | 0.4228 | Yes |
| 13 | 103241428 | SSR3 |  |  | 4441 | 1.468 | 0.4526 | Yes |
| 14 | 103228813 | SRP54 |  |  | 4484 | 1.462 | 0.4917 | Yes |
| 15 | 103234478 | LOC103234478 |  |  | 4938 | 1.408 | 0.5110 | Yes |
| 16 | 103226389 | TOMM7 |  |  | 6643 | 1.248 | 0.4699 | No |
| 17 | 103245852 | SRP14 |  |  | 6858 | 1.232 | 0.4949 | No |
| 18 | 103220202 | PEX13 |  |  | 7877 | 1.153 | 0.4817 | No |
| 19 | 103223741 | MTX3 |  |  | 8565 | 1.113 | 0.4823 | No |
| 20 | 103248787 | SRPRA |  |  | 9555 | 1.051 | 0.4676 | No |
| 21 | 103243015 | SRP68 |  |  | 10052 | 1.016 | 0.4739 | No |
| 22 | 103239946 | LOC103239946 |  |  | 12979 | 0.834 | 0.3666 | No |
| 23 | 103223888 | MTX1 |  |  | 14343 | 0.750 | 0.3267 | No |
| 24 | 103229082 | TOMM20L |  |  | 14569 | 0.735 | 0.3373 | No |
| 25 | 103227179 | LOC103227179 |  |  | 18380 | 0.452 | 0.1797 | No |
Table: GSEA details
[plain text format]

  

Fig 2: PROTEIN\_TARGETING(GO:0006605)
  
Blue-Pink O' Gram in the Space of the Analyzed GeneSet

  

Fig 3: PROTEIN\_TARGETING(GO:0006605): Random ES distribution
  
Gene set null distribution of ES for
 **PROTEIN\_TARGETING(GO:0006605)**
